# Mapping the Technological and Pharmacological Landscape of *Casearia sylvestris*: An Evidence‐Based Prospection for Wound Healing and Pain Management

**DOI:** 10.1002/cbdv.71292

**Published:** 2026-05-13

**Authors:** Luiza Gonçalves Soutier, Yasmim Parisotto de Souza Silva, Carla Suelen Gurski, Jaqueline Carneiro, Jéssica Brandão Reolon, Roberto Pontarolo, Marcel Henrique Marcondes Sari, Gabriel Blum Vestena, Weber Cláudio Francisco Nunes da Silva, Juliana Sartori Bonini, Luana Mota Ferreira

**Affiliations:** ^1^ Departamento De Farmácia Universidade Federal do Paraná ‐ UFPR Curitiba Brazil; ^2^ Programa de Pós‐graduação em Ciências Farmacêuticas Universidade Federal do Paraná ‐ UFPR Curitiba Brazil; ^3^ Departamento De Farmácia Universidade Estadual do Centro Oeste ‐ UNICENTRO Guarapuava Brazil

**Keywords:** *Casearia sylvestris*, wound healing, analgesic, patents, natural products, technological prospection

## Abstract

*Casearia sylvestris* Sw. (Salicaceae) is a South American medicinal plant traditionally used for the treatment of inflammatory conditions, pain, and wound healing, with its biological activity mainly attributed to clerodane diterpenes and flavonoids. This study assessed its therapeutic potential in wound repair and pain modulation by integrating patent landscaping with scientific evidence. A systematic search was conducted in the Orbit‐Questel database to identify relevant patent families, complemented by a literature review in PubMed, Scopus, and Web of Science. Patents and studies reporting analgesic, anti‐inflammatory, or wound‐healing activities were included. Of the 41 patent documents retrieved, eight met the inclusion criteria and were classified into three main technological strategies: (i) nanostructured sprays and biofilms containing glycolic extracts of *C. sylvestris*; (ii) synergistic herbal combinations; and (iii) innovative formulations for oral mucositis. Some patents also described the isolation of clerodane diterpenes with cytomodulatory and antitumor properties. Among 51 screened scientific articles, seven were included, providing generally supportive but heterogeneous evidence. Despite its promising potential, limitations related to chemical standardization, methodological variability, and the lack of clinical and mechanistic studies remain, highlighting the need for further research to enable safe and effective therapeutic applications.

## Graphical abstract

Technological prospection of *Casearia sylvestris* for wound healing reveals limited patent and scientific production, concentrated on topical formulations and associated analgesic and anti‐inflammatory properties. Key innovation gaps persist, including insufficient chemical and biological standardization, lack of clinical evidence, and restricted internationalization, highlighting opportunities for future translational development.

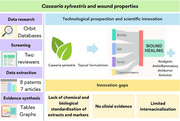

## 1 Introduction

The acute wound healing process involves a complex series of events triggered by tissue injury, including inflammation, proliferation, and remodeling, ultimately leading to the formation of an avascular scar. The initial hemostasis phase involves vasoconstriction, platelet aggregation, and fibrin formation, creating a provisional scaffold for cell migration [1, 2]. Subsequently, the inflammatory phase occurs and is characterized by the recruitment of neutrophils and monocytes, which differentiate into macrophages. These cells are responsible for phagocytosing of cellular debris and the secretion of prohealing mediators [3, 4]. In the proliferative phase, fibroblasts and myofibroblasts promote extracellular matrix deposition, angiogenesis, and granulation tissue formation, while keratinocytes migrate to restore the epidermis [5]. Finally, the remodeling phase is characterized by the reorganization of the collagen matrix, the apoptosis of inflammatory cells, and a partial restoration of tissue strength, although the skin rarely fully regains its original properties [1, 6].

Numerous local and systemic factors can delay or inhibit the healing process, leading to a chronic wound. Local factors include tissue maceration, foreign bodies, biofilm, hypoxia, ischemia, and infection. Systemic factors include diabetes, advanced age, malnutrition, and other chronic organ diseases [2]. Common clinical signs and symptoms include pain, erythema, edema, heat, and purulence. In this context, chronic wounds represent a significant healthcare burden, generating substantial financial costs and negatively affecting morbidity. Consequently, they constitute a major clinical challenge in long‐term care, with implications for both patient well‐being and healthcare systems [7].

Given these limitations, research has increasingly focused on alternative therapeutic strategies, including medicinal plants [7, 8, 9]. Several well‐established species have demonstrated significant wound‐healing potential. For example, Aloe vera is widely used for the treatment of burns and skin injuries due to its anti‐inflammatory and immunomodulatory properties, with recent reviews confirming its ability to accelerate wound healing [10]. In addition, *Curcuma longa* has been investigated for its antioxidant and anti‐inflammatory activities that support chronic wound repair. These examples highlight the relevance of medicinal plants as promising sources of therapeutic innovation [8, 9].

*Casearia sylvestris* Swartz (1860), a member of the Salicaceae family, is widely distributed in tropical and subtropical regions [11]. Its phytochemical profile is dominated by clerodane diterpenes and flavonoids, compounds associated with antimicrobial, anti‐inflammatory, analgesic, cytotoxic, and antivenom properties [12, 13, 14, 15, 16, 17]. These biological properties highlight its potential for wound healing and pain management and are supported by traditional medicinal use.

Beyond experimental evidence, patent documents represent a critical yet underexplored resource for monitoring technological advances, as they provide insights into formulations, dosage forms, and therapeutic applications [18]. In parallel, scientific publications remain the primary source of evidence regarding pharmacological effects, mechanisms of action, and clinical relevance. By integrating both perspectives, this study aims to analyze the patent landscape and evaluate the scientific literature to comprehensively elucidate the therapeutic potential of *C. sylvestris* as a wound‐healing and analgesic agent. This integrated approach, combining patent landscaping with a systematic review of the literature, has been successfully applied to other medicinal plants [9, 19]. Such precedents reinforce the relevance of the present strategy for comprehensively capturing the therapeutic potential of *C. sylvestris*.

## 2 Methodology

The present technological prospecting aimed to conduct a comprehensive ‘state of the art search,′ to elucidate the current technological landscape regarding the therapeutic applications of *C. sylvestris* as a wound‐healing agent and analgesic agent. An exploratory documentary patent research, incorporating both qualitative and quantitative approaches, was performed based on patent families [9]. The search was conducted in July 2023 using the Orbit‐Questel database. A combination of relevant keywords was applied using Boolean to ensure specificity, without restricting the publication year. The term “*Casearia sylvestris*” was used alone or combined (AND) with the following descriptors: “painkiller”, “analgesic”, “cicatrizant”, “scarifier”, “scar”, “wound healing”, “anodyne”, “antinociceptive”, “cicatrix”, “cicatrization”, “wound closure”, “anti‐inflammatory”, “anti‐inflammator”, and “antiinflammator” under the “Easy Search” field. Patents demonstrating the use of *C. sylvestris* for analgesic or wound‐healing purposes were included. Documents not related to these pharmacological activities were excluded. The full patent list, search strategies, and raw data tables are provided in the Supporting Information (Tables S1, S2, and S3). The data used for figure generation were extracted and processed from the Orbit‐Questel platform, for which a limited number of licenses are made available free of charge for academic use, including access provided to the PROFNIT (Professional Master's Program in Intellectual Property and Technology Transfer for Innovation) program, all figures were produced by the authors based on the obtained data.

Given the limited number of relevant patent records, a systematic investigation of the scientific literature addressing the therapeutic potential of *C. sylvestris* for wound healing and pain relief was conducted in March 2025. Searches were performed in PubMed, Scopus, and Web of Science databases. Descriptors were combined with the Boolean operators “OR” and “AND”, without time or language restrictions to answer the research question: “**What is the therapeutic potential of *Casearia sylvestris* in promoting wound healing and pain relief?**”. Search results from each database were exported to the Rayyan online platform (free version; Rayyan Systems, Inc.) for screening. Two independent reviewers conducted the initial screening, and disagreements were resolved through consultation with a third reviewer. Duplicate records and studies outside the predefined scope were removed. Full‐text articles were subsequently retrieved and assessed for eligibility according to the established inclusion criteria. Studies reporting the use of *C. sylvestris* for wound healing or analgesic purposes were considered eligible. Articles that did not address these specific pharmacological activities, as well as opinion articles and review papers, were excluded.

Following retrieval and screening, data extraction was performed for both patents and scientific articles. For patent documents, structured data, including publication year, geographical coverage, applicants, and associated institutions, were collected. Additionally, unstructured data, such as pharmaceutical formulations, therapeutic applications, and technological approaches, was manually extracted through full‐text analysis and categorized according to technological domain and therapeutic indication.

For the scientific articles, detailed data were extracted regarding study design, plant part used, extraction method, dosage form, route of administration, pharmacological activity, and primary experimental outcomes. All extracted information was organized into spreadsheets to enable both quantitative assessment and qualitative synthesis. This approach helped with the identification of technological trends, experimental evidence, and therapeutic applications of *C. sylvestris* in wound healing and pain management.

Finally, an evidence map was constructed to integrate and visually represent the extracted data. Descriptive statistical analysis was performed to determine the frequency and distribution of formulations, pharmacological applications, and innovation types. A Sankey diagram was subsequently developed to illustrate the relationships among technological approaches, biological activities, and geographical markets. This combined analytical strategy enabled the identification of key research patterns and translational evidence gaps in the development of *C. sylvestris*‐based products.

## 3 Results and Discussion

### 3.1 Patent Analysis

Patents represent a key source of technological knowledge. Exploring patent documents enables the identification of novelty, analysis of technological trends, detection of gaps and innovation hotspots, anticipation of future developments, and support for strategic technology planning [20]. Orbit (Questel) is a proprietary platform that integrates extensive intellectual property databases and provides an advanced keyword‐based search interface, enabling efficient retrieval of relevant patent documents.

The patent screening followed predefined inclusion and exclusion criteria. Documents were included when they were published within the defined study period, explicitly referred to *C. sylvestris*, and addressed technological developments related to therapeutic applications, including wound healing, pain management, anti‐inflammatory activity, extraction processes, pharmaceutical formulations, or bioactive compound isolation. Exclusion criteria included duplicate records, documents not specifically related to *C. sylvestris*, patents lacking technological relevance to the study objectives, and records with insufficient technical information.

**FIGURE 1:**
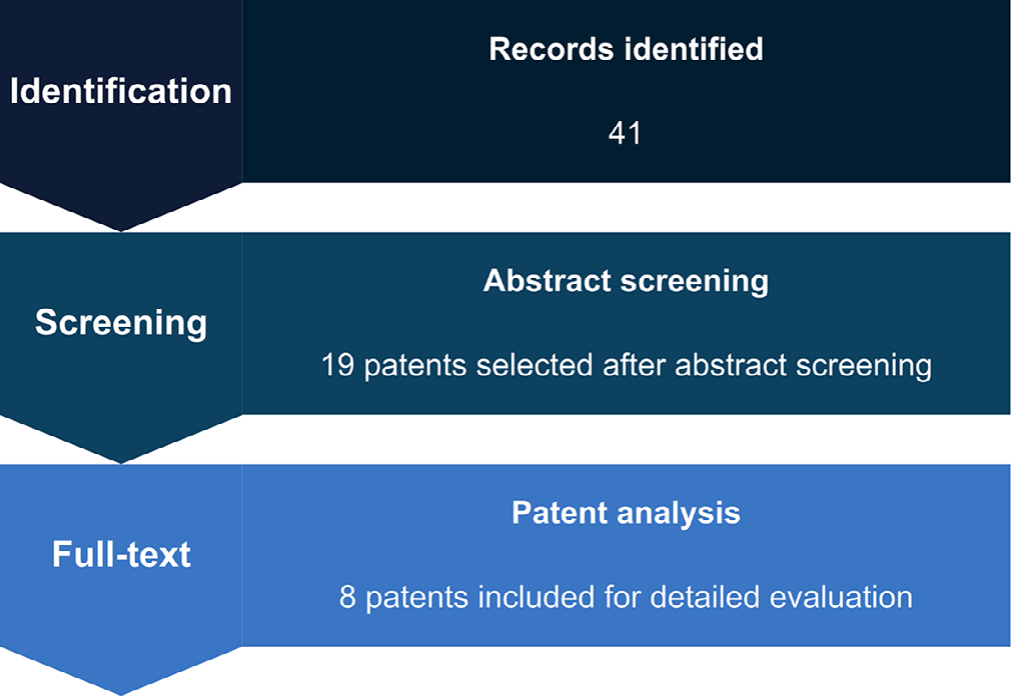
Systematic patent search flowchart.

The initial search identified 41 patent records. After duplicate removal and title/abstract screening, 19 patents were considered potentially eligible. These documents were subsequently subjected to full‐text assessment, resulting in eight patents that met all inclusion criteria and were included in the analysis and discussion. This structured screening process ensured transparency and alignment between the search strategy, methodological framework, and the final dataset used for qualitative evaluation (Figure 1).

**FIGURE 2:**
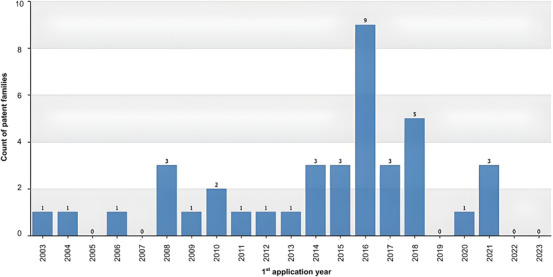
Annual distribution of *Casearia sylvestris* patent families (Orbit‐Questel, 2023).

Analysis of the annual distribution of *C. sylvestris* patent families (*n* = 41) revealed a prolonged period of low filing activity, with only a small number of applications submitted between 2003 and 2013 (Figure 2). A marked increase was observed in 2016 (nine filings), followed by another peak in 2018 (five filings), and a subsequent decline in the following years (Figure 2). This temporal distribution suggests a period of intensified innovation activity during the mid‐2010s, contrasting with the relatively sparse patenting behavior observed previously.

The reduction in patent filings after 2019 may be partially associated with broader disruptions affecting research, development, and intellectual property activities globally, including the COVID‐19 pandemic, which impacted laboratory workflows, funding priorities, and technology transfer processes. However, this interpretation should be considered as a plausible contextual factor rather than a direct causal explanation [21].

The increase in filings during this period may be associated with intensified scientific interest in the pharmacological properties of *C. sylvestris*, particularly its anti‐inflammatory, wound‐healing, and antitumor potential, which have been increasingly investigated in experimental studies [22]. Additionally, the enactment of the Brazilian Biodiversity Law (Law N° 13,123/2015) in 2016 may have contributed to greater formalization and protection of innovations derived from native biodiversity, potentially influencing the surge in patent applications during this period [23].

The geographical distribution of patents related to *C. sylvestris* demonstrates a clear predominance in Latin America, with Brazil (10 filings) and Mexico (7 filings) representing the largest markets (Figure S1A,B). This pattern reflects both the natural distribution of the species in these countries and the significant strategic interest in protecting innovations derived from its traditional uses and pharmacological potential [24]. Patent activity in other regions, including the United States, Europe, and Asia, indicates a gradual international expansion of interest. This dispersion may reflect efforts to secure intellectual property protection in high‐value markets, thereby supporting competitive positioning should the species gain broader recognition in global pharmaceutical or cosmeceutical development.

Analysis of the legal status of *C. sylvestris* patents (*n* = 41) showed that 34.1% have already been granted (Figure S2), indicating that a substantial proportion of inventions successfully completed examination and obtained intellectual property protection. A further 19.5% remain pending, suggesting ongoing technological activity and potential future market protection.

In addition, 19.5% of patents were revoked, possibly due to failure to meet requirements related to novelty, inventive step, or regulatory compliance. By contrast, 22.0% lapsed, frequently as a result of nonpayment of maintenance fees, which may indicate limited commercial exploitation of certain filings. Only 4.9% of patents have expired, suggesting that most inventions are relatively recent, consistent with the increase in filing activity observed after 2015. These findings indicate that a substantial proportion of inventions have successfully progressed through examination and obtained intellectual property protection, while a relevant share (19.5%) remains pending, reflecting ongoing technological activity and potential future market protection. Overall, the results suggest that *C. sylvestris* represents an active field of technological development with potential for further expansion. The predominance of valid and pending applications suggests ongoing innovation efforts, whereas revoked and lapsed patents reflect the selective consolidation of more viable technological approaches.

**FIGURE 3:**
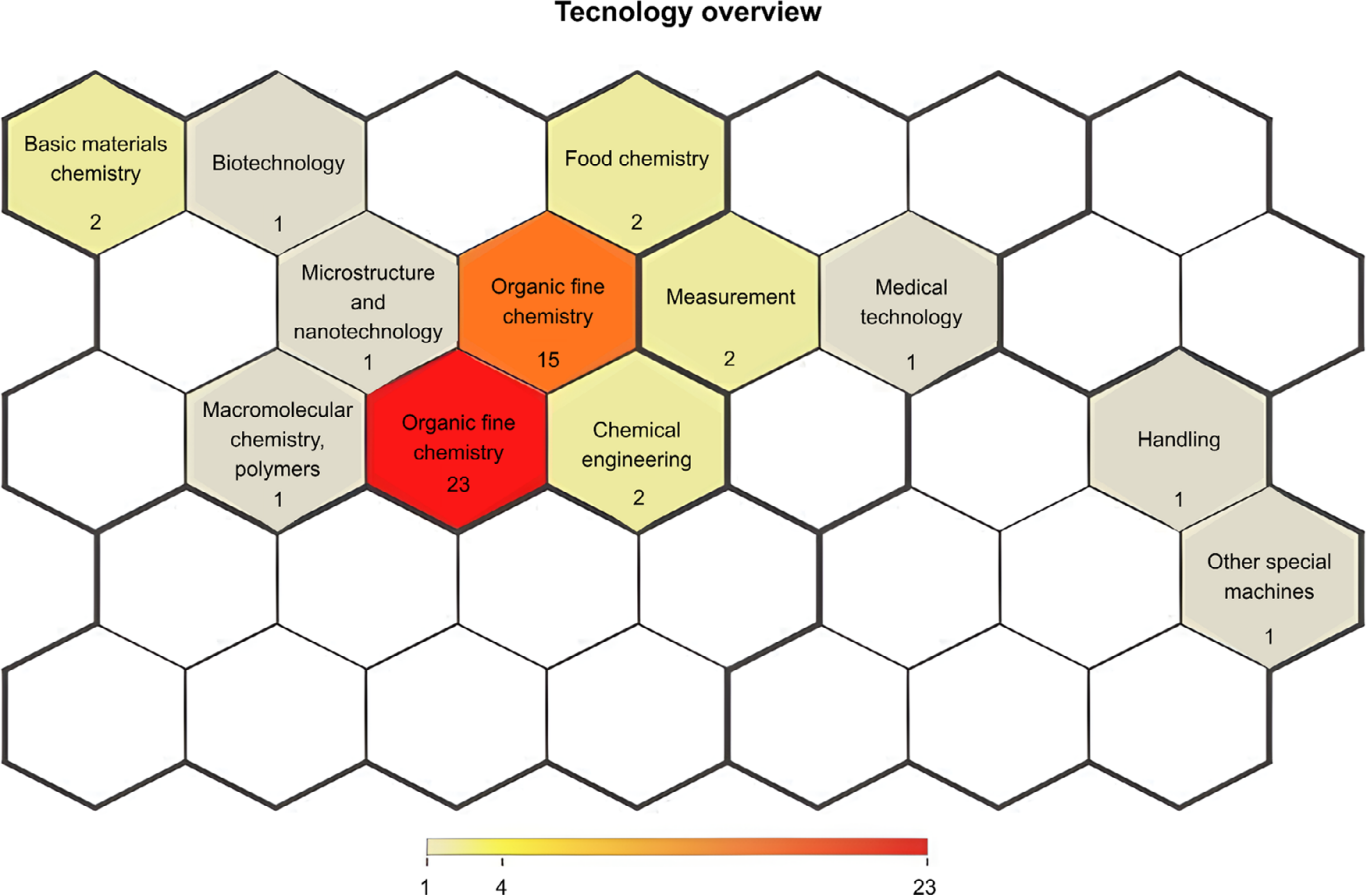
Patent families by technological domain (Orbit‐Questel, 2023).

As shown in Figure 3, patent activity related to *C. sylvestris* is concentrated in a limited number of technological domains, with Pharmaceuticals (23 filings) and Organic Fine Chemistry (15 filings) accounting for most applications. This distribution reflects a clear orientation toward medicinal product development and phytochemical investigation. The data indicate a focus on drug product development, including dosage forms and delivery systems, as well as on the identification, isolation, and structural modification of bioactive compounds, particularly clerodane diterpenes and flavonoids, which are associated with the pharmacological properties of the species [17, 25]. In contrast, domains such as Food chemistry, Chemical engineering, and Basic materials chemistry appear only sporadically, suggesting limited diversification of innovation pathways.

The predominance of pharmaceutical filings is consistent with the experimental literature reporting anti‐inflammatory, analgesic, and wound‐healing effects of *C. sylvestris* extracts in different biological models [13, 22, 26, 27, 28]. From a technology monitoring perspective, this concentration reflects patterns observed in other biodiversity‐based innovations, in which research efforts are primarily directed toward chemical characterization and formulation, while applications in biotechnology and advanced materials remain less explored.

Together, these findings suggest clear opportunities for portfolio expansion, particularly in biotechnology, including standardized extracts, biotransformation, and omics‐guided target discovery, as well as in biomaterials for wound care, such as bioactive films and advanced dressings, which remain underrepresented in the current technological landscape.

**FIGURE 4:**
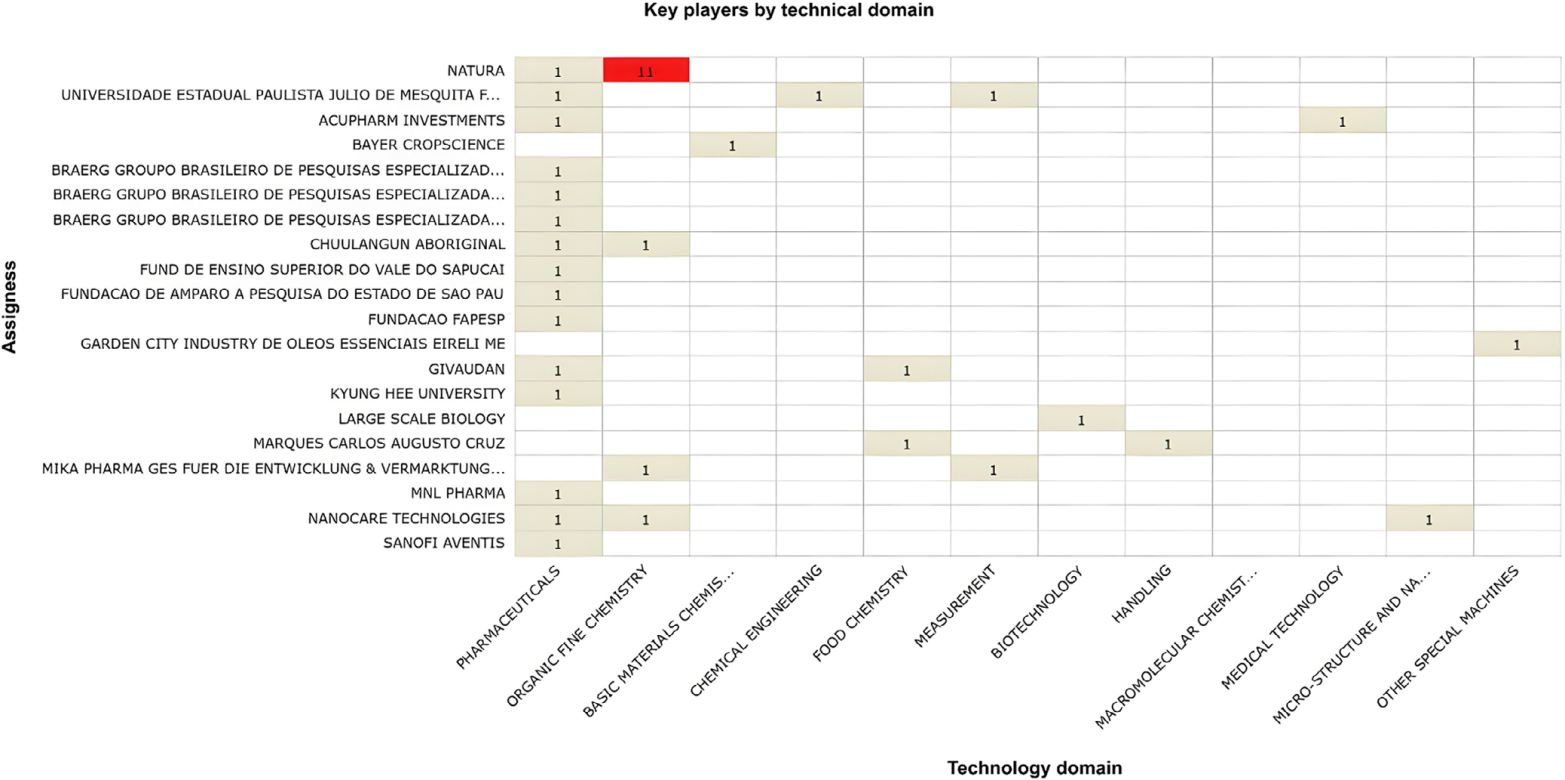
Distribution of patent assignees by technological domain (Orbit‐Questel, 2023).

The distribution of patent assignees further reinforces this concentration. As shown in Figure 4, innovation involving *C. sylvestris* is largely concentrated among a limited number of institutions and companies, with a small group accounting for most filings. This pattern indicates a restricted diversification of technological ownership and suggests that innovation in this field remains centralized rather than broadly distributed across multiple actors.

Natura, a Brazilian multinational, stands out as the top assignee, with patents mainly in organic fine chemistry. This reflects its long‐term strategy of incorporating biodiversity into product development, particularly in cosmetics and pharmaceuticals applications. The emphasis on biodiversity‐based innovation highlights Brazil's natural resource base, but also underscores the challenge of converting scientific knowledge into marketable products [29]. As noted by Braga (2021), despite the growth in research on Brazilian medicinal plants, this expansion has not been matched by a proportional increase in commercially viable innovations, suggesting structural barriers in technology transfer and industrial scaling [30].

Academic institutions, such as Universidade Estadual Paulista (UNESP), also contribute to the patent landscape, although with fewer filings and a broader distribution across technological domains. This pattern aligns with the exploratory role typically played by universities, whose research often precedes industrial application [31]. International companies, including Bayer and Grünenthal, appear only sporadically, which may reflect limited or opportunistic engagement rather than sustained investment in this field.

**FIGURE 5:**
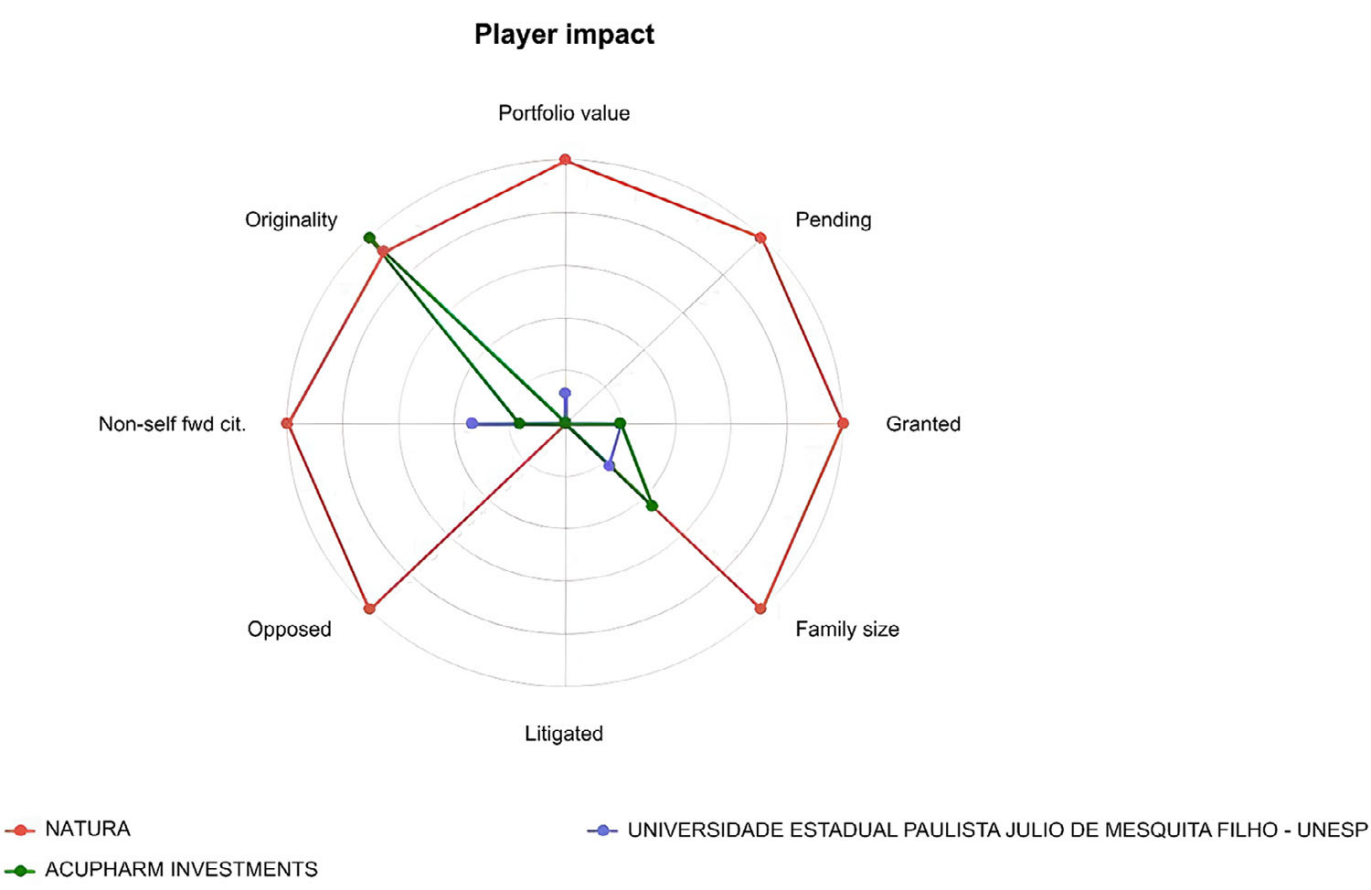
Radar analysis of the three main assignees (Orbit‐Questel, 2023).

The comparative innovation profile of the three most active assignees is presented in Figure 5. The radar analysis highlights differences in portfolio strength and technological positioning, reinforcing the asymmetric distribution of innovation capacity observed in the patent landscape.

Natura demonstrates the broadest technological footprint, with higher values in portfolio value, granted and pending applications, and family size, indicating a mature yet expanding portfolio protected across multiple jurisdictions. This profile aligns with sustained research and development, as well as a strategy of international coverage for biodiversity‐derived products [29]. Litigated and opposed remain low across players, suggesting a field that is technologically active but not highly contentious, while non‐self forward citations for Natura point to some external knowledge uptake. Acupharm Investments show a pronounced peak in originality, a metric that captures the diversity of prior‐art classes cited and the recombination of technological knowledge; such spikes are often seen in smaller portfolios exploring distinctive chemical/technological space [20]. UNESP exhibits modest values across axes, reflecting an academic profile with fewer families and lower portfolio value, but contributing to upstream knowledge that can later be appropriated by industry [20].

Our initial search and screening process identified eight patents suitable for use in wound healing and pain relief. These documents highlight the most relevant technological advancements related to *C. sylvestris*, including various approaches such as extraction methods, pharmaceutical formulations, the isolation of bioactive compounds, and therapeutic applications, particularly in the contexts of wound healing, anti‐inflammatory, and analgesic effects. Table 1 provides a summary of the selected patents, including their publication numbers and translated titles.

**TABLE 1:** Patent search results.

Seq.	Publication number	Title	Main findings
01	BRPI0602094	Medicinal composition based on *Casearia sylvestris* and use of medicinal composition based on *C. sylvestris*	Herbal and homeopathic formulations (creams, gels, sprays) for treating herpes labialis. Demonstrated reduced healing time and lower cost compared to conventional antivirals.
02	BRPI0306167	Process of obtaining extracts and active fractions of *C. sylvestris* and their uses	Describes extraction processes and fractionation to obtain standardized active fractions—applications in gastrointestinal disorders and as antiulcer, anti‐inflammatory, and healing agents.
03	BR102018008368	Mouthwash with a solution based on Melaleuca alternifolia and *C. sylvestris* for the prevention and treatment of mucositis in patients undergoing chemotherapy	Mouthwash with 8% *C. sylvestris* + 7% tea tree oil. Developed to prevent and treat oral mucositis in cancer patients undergoing chemotherapy. Clinical application in supportive oncology care.
04	BRPI0900645	Extracts, active fractions and/or isolated compounds of *C. sylvestris*, pharmaceutical formulations and their uses	Isolation of bioactive diterpenes (casearins B, U, caseargrevin F). Applications in gastrointestinal disorders, especially gastroduodenal ulcers. Provides pharmaceutical forms for oral and parenteral administration.
05	BR102018071621A2	Effect of glycolic extract of *C. sylvestris* Swartz (guaçatonga) on wound repair in rats	Nanostructured glycolic extract formulated as a spray or chitosan biofilm. Demonstrated accelerated wound healing in animal models (rats).
06	BR102014008978A2	Medicinal composition with antibiotic, anti‐inflammatory, and healing action	Synergistic topical composition of *Matricaria recutita*, *Psidium guajava*, *Plantago major*, and optionally *C. sylvestris*. Applied as a biofilm, liquid, or solid dosage form. Indicated for burns and chronic wounds.
07	BRPI0805322A2	Compounds with cytomodulatory action, formulations containing them, and processes for their preparation	Isolation of Casearin X (clerodane diterpene). Shows antitumor activity through modulation of proliferative disorders. Potential use in oncology.
08	WO2021191811	A novel wound gel composition	Gel/biofilm containing synergistic extracts (Matricaria, Psidium, Plantago, optionally *C. sylvestris*). Antibacterial, anti‐inflammatory, and wound‐healing properties. International extension of BR102014008978.

An analysis of the set of patents involving *C. sylvestris* highlights three main areas of technological development: (i) topical formulations for wound healing, such as sprays or chitosan biofilms containing the plant's glycolic extract; (ii) synergistic combinations with other plant extracts, such as *Matricaria recutita, Psidium guajava, and Plantago major*, resulting in antibacterial, anti‐inflammatory, and healing biofilms and gels; and (iii) innovative solutions for oral mucositis, with the combined use of *C. sylvestris* and tea tree oil, intended for patients undergoing chemotherapy. These patents demonstrate the species' relevance in contexts of high inflammatory burden and pain, reinforcing its potential as a multifunctional phytotherapeutic.

Although modern formulations like nanostructured systems and biofilms have advanced, most lack strong clinical evidence. This trend is also seen in other technological approaches involving different plant families [19]. The exception is a patent for mouthwash used to treat mucositis, which reports use in cancer patients but is not supported by randomized clinical trials. Likewise, patents on isolated compounds, such as casein X, exhibit promising antitumor activity but remain in the preclinical stage, requiring comprehensive pharmacological and toxicological validation [32].

Another relevant point is the lack of standardization. Patents, such as BR200900645, describe percentage ranges of active ingredients for extracts and fractions, but do not establish analytical criteria comparable to those in compendial monographs, which limits reproducibility and clinical translation [33]. This lack of quality restrictions also makes regulation more susceptible, especially in international markets.

Finally, it should be noted that claims often mention synergistic and multipurpose effects (healing, anti‐inflammatory, antimicrobial, and analgesic), but few provide elucidated mechanisms of action [34, 35, 36, 37]. Cellular and molecular assays targeting inflammatory markers, cell migration, or extracellular matrix deposition could strengthen the originality and competitiveness of inventions.

Overall, the validated patents highlight a significant emphasis on using *C. sylvestris* in supportive and healing treatments. However, they encounter regulatory and scientific hurdles that require clinical trials, chemical standardization, and mechanistic research. These challenges are common to other plants as well products [19]. These elements should guide future development and the consolidation of phytotherapy into innovative products for clinical use.

### 3.2 Scientific Innovation

**FIGURE 6:**
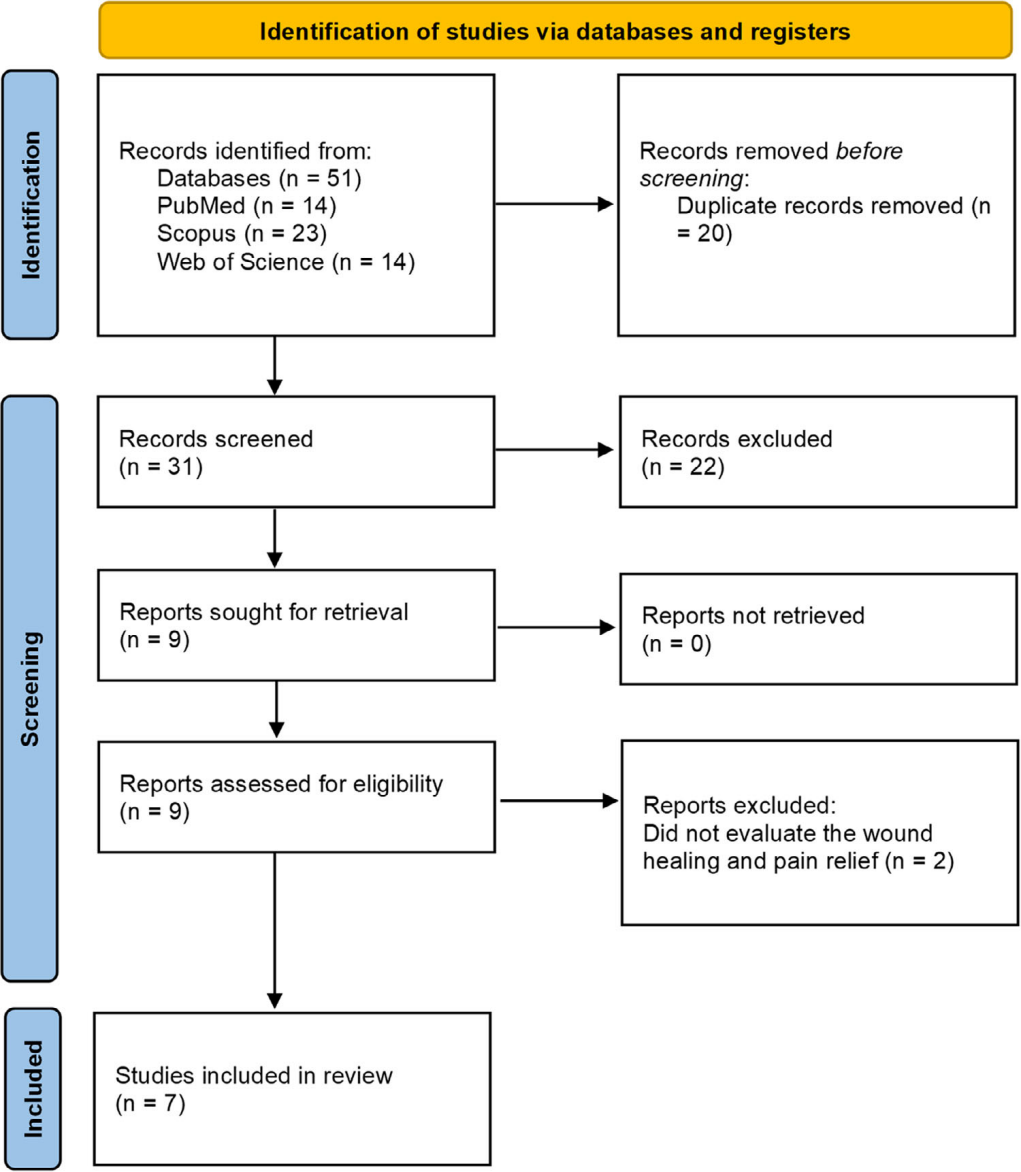
Scoping review flow diagram (PRISMA‐ScR) [38].

After the search, 51 articles were identified, including 14 in PubMed, 23 in Scopus, and 14 in the Web of Science. After applying the eligibility criteria, which required that studies use *C. sylvestris*, incorporate the plant into a pharmaceutical formulation, and evaluate its wound‐healing and anti‐inflammatory potential, only seven studies met the requirements and were selected for characterization and discussion in this research (Figure 6). This progressive reduction from the initial pool to a small number of eligible studies reflects the limited volume of structured experimental evidence available on *C. sylvestris*, despite its technological relevance observed in the patent landscape.

The characteristics of the selected studies are summarized in Table 2, and the complete data extraction is provided in the Supporting Information (Table S4). These studies investigated the analgesic and wound‐healing effects of *C. sylvestris*. The oldest article was published in 1991, with two articles published between 2005 and 2007, five articles published between 2012 and 2017, and the most recent article published in 2021. All seven articles are Brazilian, likely due to the widespread distribution of the plant in tropical and subtropical regions, it is reasonable to assume that most scientific research is conducted in locations where the plant is most abundant [24]. This greater incentive to research the plants′ possible pharmacological properties within Brazilian territory is also evidenced by the fact that the majority of published patents originate from Brazil.

**TABLE 2:** Description of studies found in the search for scientific innovation.

Reference	Dosage form	Pharmacological application
Napolitano, 2005	Crude plant extract	Anti‐inflammatory
De Mattos, 2007	Hidroalcoholic extract	Antinociceptive
Lipinski, 2012	Gel	Wound healing
Trecco, 2014	Latex membranes	Wound healing and anti‐inflammatory
De Campos, 2015	Biofilm and liquid formulation	Healing process of burn injuries
Piovezan, 2017	Ointment	Treatment of chronic post‐ischemia (CPIP)
Dezena, 2021	Tincture	Healing of tissues

The wound‐healing potential of *C. sylvestris* has been investigated using different extraction methods, formulations, and experimental models, yielding heterogeneous results that appear to depend strongly on preparation type and route of administration.

In a study conducted by Lipinski et al. (2012), decoctions prepared by boiling 100 g of plant material per liter of water were incorporated into a carboxymethyl cellulose gel and applied topically to experimentally induced skin wounds in Puruna heifers for 17 days. Despite the traditional use of *C. sylvestris* for wound care, the treatment demonstrated limited efficacy, with no significant effects on wound contraction or histological parameters [39].

In contrast, studies employing hydroalcoholic extracts reported more favorable outcomes. Piovezan et al. (2017) used a 70% hydroethanolic leaf extract, administered both topically (2.5% lanolin‐based ointment) and orally in rodents. This approach resulted in enhanced wound contraction, increased collagen deposition, fibroblast proliferation, reduced inflammatory cell infiltration, and systemic anti‐inflammatory effects, including decreased exudate formation and granulomatous tissue in the cotton pellet‐induced granuloma model [27]. Similarly, De Campos et al. (2015) evaluated hydroalcoholic leaf extracts incorporated into liquid formulations and biofilm dressings in a second‐degree scald burn model in rats. Macroscopic and histopathological analyses demonstrated improved skin regeneration, likely associated with modulation of inflammatory mediators [22].

However, not all hydroalcoholic preparations yielded robust tissue effects. Dezena et al. (2021) assessed 10% tinctures prepared by percolation with 70% ethanol and observed limited structural impact on collagen fibers, although a reduction in elastic fibers was noted after 48 h of incubation. The absence of significant proteolytic activity on collagen suggests that compositional differences, particularly the relative abundance of diterpenes and gallic acid derivatives and the apparent lack of tannins, may influence tissue remodeling capacity [28].

Beyond wound contraction models, analgesic and anti‐inflammatory properties have also been demonstrated in experimental systems that may indirectly support wound‐healing mechanisms. Ruppelt (1991) reported significant analgesic activity, evidenced by reduced contortions in mice, and moderate anti‐inflammatory effects through partial inhibition of Evans blue dye diffusion [26]. Likewise, De Mattos et al. (2007) demonstrated dose‐dependent antinociceptive effects of hydroalcoholic crude extract in inflammatory pain models, with high doses producing responses comparable to morphine in hot plate tests and to indomethacin in writhing assays. These findings suggest combined modulation of inflammatory mediators and possible involvement of opioidergic pathways [13].

Finally, formulation strategy also appears to influence therapeutic applicability. Trecco et al. (2014) developed a controlled‐release system using natural latex membranes containing ethanolic leaf extracts. A biphasic release profile was observed, with pH‐dependent kinetics affecting the availability of phenolic compounds over a 10‐day period, supporting the potential of modulated delivery systems in wound treatment contexts [40].

Collectively, these findings indicate that the therapeutic performance of *C. sylvestris* in wound‐healing applications varies considerably according to extraction method, solvent system, dosage form, and route of administration. Hydroalcoholic preparations and structured delivery systems appear to produce more consistent biological responses than aqueous decoctions, highlighting the critical role of formulation design in determining pharmacological outcomes.

## 4 Evidence Gap Map

**FIGURE 7:**
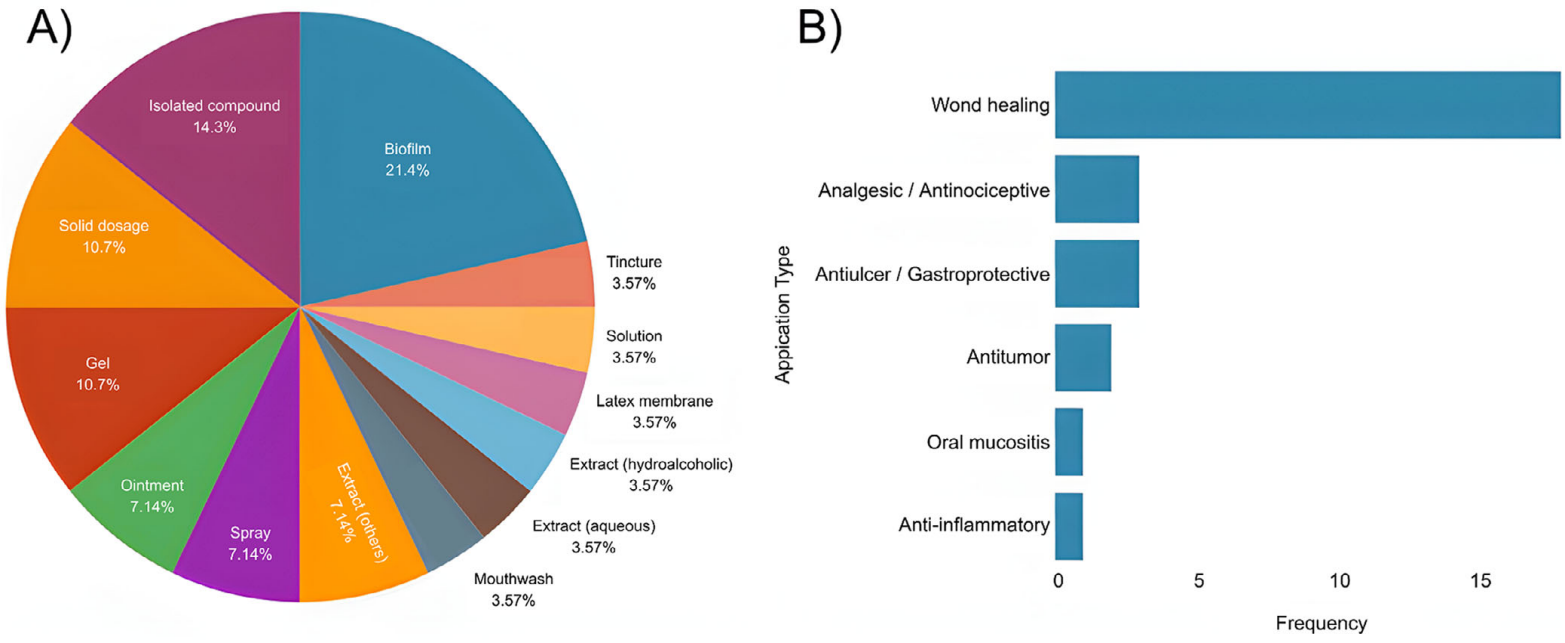
Technological (A) and pharmacological (B) distribution of *Casearia sylvestris* innovations.

The evidence map integrates the technological and pharmacological dimensions of *C. sylvestris* innovation, illustrating how research and development efforts have been distributed across formulation types, therapeutic applications, and stages of innovation. As shown in Figure 7A, biofilms (21.4%), isolated compounds (14.3%), gels (10.7%), and solid dosage forms (10.7%) represent the most frequently explored systems. This pattern reflects a predominance of topical and semisolid formulations, which is consistent with the traditional use of *C. sylvestris* in wound healing and inflammatory conditions [22, 27, 39, 40]. Although different technological matrices have been investigated, most developments remain at experimental or preclinical stages, indicating limited translational progression.

Figure 7B further demonstrates that wound healing (71.4%) is the primary therapeutic focus across both patents and scientific publications. Other reported applications, including analgesic, antiulcer, antitumor, and anti‐inflammatory activities, appear less frequently and are generally supported by preliminary experimental data. This distribution suggests that, despite the diversity of claimed pharmacological effects, innovation and scientific validation remain concentrated in a narrow therapeutic domain.

The percentages presented in Figure 7 were calculated based on the combined dataset of selected patents and eligible scientific articles.

**FIGURE 8:**
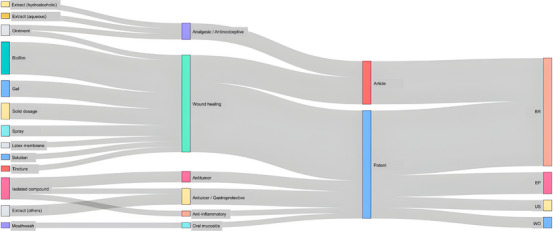
Sankey diagram of technological and therapeutic distribution of *Casearia sylvestris* innovations.

The integrated relationship between technological classification and therapeutic application is summarized in Figure 8. The Sankey diagram shows that innovation involving *C. sylvestris* is predominantly directed toward topical formulations for wound healing, particularly gels, biofilms, and sprays. This pattern is consistent with the concentration of preclinical studies investigating regenerative and anti‐inflammatory effects.

In contrast, applications related to analgesic, anti‐inflammatory, and antitumor activities appear less frequently and are supported by a smaller number of technological developments. This distribution reinforces the observation that, although multiple pharmacological effects have been reported, innovation efforts remain largely focused on a single therapeutic axis.

The overlap between patents and scientific articles shows that most innovations are still in early or preclinical stages, with limited evidence of clinical testing or market launch. Patent filings indicate diversification in pharmaceutical forms, including nanostructured sprays, chitosan biofilms, and polyherbal mixtures, but only one (a mouthwash for oral mucositis) has been tested in humans [32, 35, 36, 37]. Most scientific studies use animal models [13, 22, 26, 27, 28], and none employ standardized extracts or measure bioactive components, which hampers comparison and reproducibility. This gap between technological development and pharmacological validation is a significant obstacle to advancing *C. sylvestris* toward practical, clinical use.

The network structure of the Sankey also highlights an asymmetrical difference between innovation type and geographical distribution. Most patents and all scientific articles originated in Brazil, reflecting strong academic interest but limited internationalization and industrial uptake. The scarcity of filings in Europe, the United States, and Asia suggests underexploited opportunities for global protection and commercialization of biodiversity‐based technologies. The reliance on natural or crude extracts instead of purified or standardized fractions highlights a dependence on empirical formulations. This approach contrasts with current regulatory standards for botanical drugs [41].

From a mechanistic perspective, another evidence gap concerns the lack of studies investigating molecular pathways involved in wound healing and pain modulation by *C. sylvestris*. Although some patents claim antioxidant, antimicrobial, and anti‐inflammatory mechanisms, few provide experimental substantiation. The absence of pharmacodynamic and pharmacokinetic characterization, particularly of clerodane diterpenes and flavonoids, limits the ability to define safe and efficacious dose ranges or to establish quality specifications [41, 42]

Overall, the evidence map reveals a fragmented innovation ecosystem: patents are largely exploratory and localized, whereas scientific research, though growing, has not yet translated into standardized, clinically tested, or commercially viable products. Bridging these gaps requires a coordinated strategy encompassing: (I) chemical and biological standardization of extracts and markers; (II) advanced preclinical and clinical trials demonstrating efficacy and safety; (III) investment in biotechnological platforms for compound isolation, nanoformulation, and delivery systems; (IV) stronger academic–industry partnerships to ensure continuity from discovery to market.

## 5 Conclusion

This study provides an integrated overview of the technological and scientific landscape of *C. sylvestris* in wound healing and pain management by combining patent analysis with evidence mapping of the scientific literature. The findings demonstrate that innovation is predominantly focused on topical pharmaceutical formulations and exploratory extraction strategies, while mechanistic studies, standardization approaches, and clinical validation remain limited.

The combined analysis revealed a fragmented innovation pipeline, characterized by active patenting activity and growing experimental evidence, yet with restricted translational progression toward clinically validated products. This gap highlights the need for stronger alignment between phytochemical characterization, technological development, and pharmacological validation.

From a scientific perspective, this work contributes by systematically linking technological intelligence with experimental evidence, enabling the identification of priority areas for research and development involving biodiversity‐derived therapeutics. Practically, the results emphasize challenges related to extract standardization, regulatory requirements, and scalability, which represent critical barriers to industrial and clinical application.

Future research should prioritize standardized extracts, mechanistic investigations, advanced preclinical models, and clinical studies capable of supporting regulatory approval. In addition, investment in biotechnological platforms, nanoformulation strategies, and academic–industry collaboration may accelerate the translation of *C. sylvestris* into innovative therapeutic products.

Overall, *C. sylvestris* represents a promising natural source for regenerative and analgesic applications; however, coordinated multidisciplinary efforts are required to bridge the gap between ethnopharmacological knowledge, technological development, and market implementation.

## Author Contributions

**Luiza Gonçalves Soutier**: Methodology, data curation, investigation, writing – original draft, Writing – review and editing, visualization, conceptualization. **Yasmim Parisotto de Souza Silva**: Methodology, data curation, investigation, review, writing – original draft, writing – review and editing, visualization, conceptualization. **Gabriel Blum Vestena**: Methodology, data curation, investigation, writing – review and editing. **Carla Suelen Gurski**: Methodology, data curation, investigation, reading and review. **Jaqueline Carneiro**: Methodology, data curation, investigation, reading and review. **Marcel Henrique Marcondes Sari**: Conceptualization, data curation, reading and review. **Roberto Pontarolo**: Conceptualization, reading and review, validation, supervision. **Weber Cláudio Francisco Nunes da Silva**: Conceptualization, methodology, data curation, investigation, writing– review and editing. **Juliana Sartori Bonini**: Conceptualization, methodology, data curation, investigation, writing – review and editing, validation, supervision. **Luana Mota Ferreira**: Conceptualization, methodology, data curation, investigation, writing – review and editing, validation, supervision.

## Funding

The authors are grateful to Fundação Araucária—PR (TED 27/23) for the financial support.

## Conflicts of Interest

The authors declare no conflict of interest.

## Supporting information




**Supporting File 1**: cbdv71292‐sup‐0001‐SuppMat.docx

## Data Availability

Data sharing not applicable to this article as no datasets were generated or analysed during the current study.
