# Supplementary material for: Mapping the Technological and Pharmacological Landscape of Casearia sylvestris: An Evidence‐Based Prospection for Wound Healing and Pain Management
Source: Chem Biodivers. 2026 May 13;23:e71292. doi: 10.1002/cbdv.71292 (PMC13170992; doi:10.1002/cbdv.71292)
Supplement: Supplementary file 1 — Supporting File 1: cbdv71292‐sup‐0001‐SuppMat.docx [file CBDV-23-e71292-s001.docx]

**Mapping the Technological and Pharmacological Landscape of *Casearia sylvestris*: An Evidence-Based Prospection for Wound Healing and Pain Management**

Luiza Gonçalves Soutier^a^•, Yasmim Parisotto de Souza Silva^a^•, Carla Suelen Gurski^c^, Jaqueline Carneiro^c^, Jéssica Brandão Reolon^b^, Roberto Pontarolo^a,c^, Marcel Henrique Marcondes Sari^c^, Gabriel Blum Vestena^b^, Weber Cláudio Francisco Nunes da Silva^b^, Juliana Sartori Bonini^b^, Luana Mota Ferreira^a,c^*.

*•These authors contributed equally to this study.*

^a^ Departamento de Farmácia, Universidade Federal do Paraná - UFPR, Curitiba, Brasil

^b^ Departamento de Farmácia, Universidade Estadual do Centro Oeste - UNICENTRO, Guarapuava, Brasil

^c^ Programa de Pós-graduação em Ciências Farmacêuticas, Universidade Federal do Paraná - UFPR, Curitiba, Brasil.

* Departamento de Farmácia, Universidade Federal do Paraná, Curitiba, 80210-170, Brazil. Email: [luanamota@ufpr.br](mailto:luanamota@ufpr.br). Phone: +55 41 3360 4095

**Supplementary Material – Figures**

**
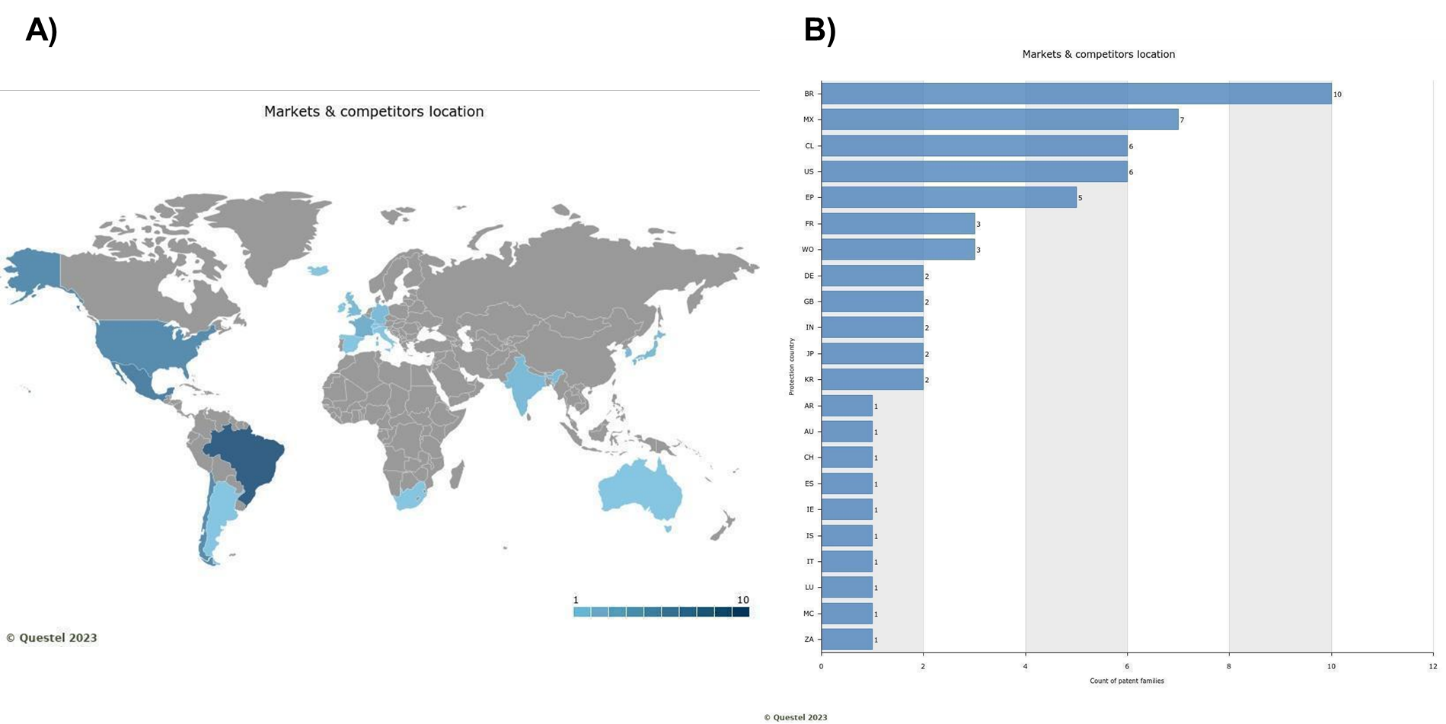
**

**Figure S1.** Geographical distribution of *Casearia sylvestris* patent families by market. **(A)** World map of filings. **(B)** Number of patent families per country. Source: Orbit (2023).


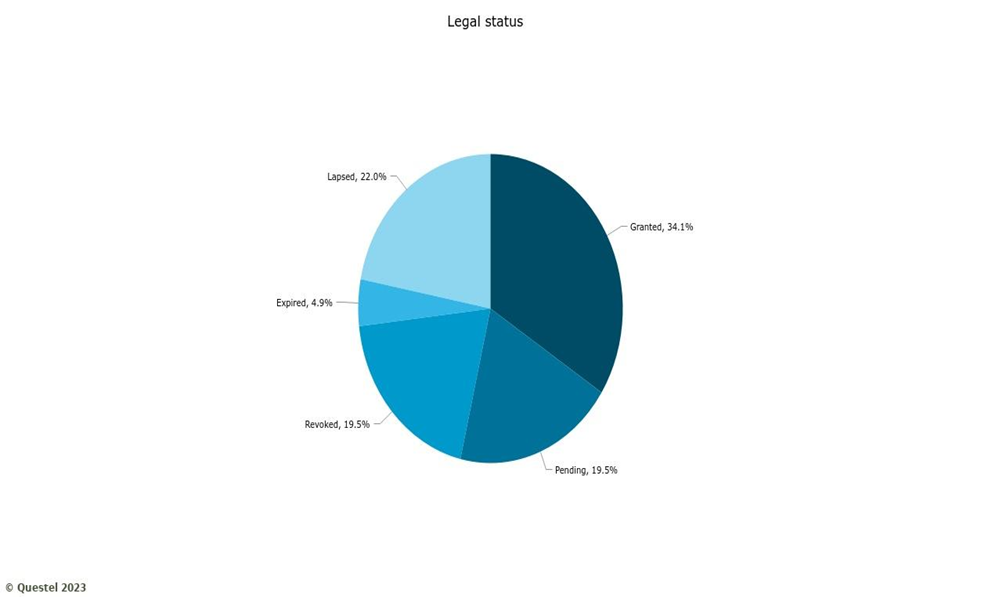


**Figure S2.** Legal status. Source: Orbit (2023).

**Table S1.** Patent search results

| **Term combination** | **Results** |
| --- | --- |
| *Casearia sylvestris* | 41 |
| *Casearia sylvestris* AND painkiller | 0 |
| *Casearia sylvestris* AND analgesic | 4 |
| *Casearia sylvestris* AND cicatrizant | 0 |
| *Casearia sylvestris* AND scarifier | 0 |
| *Casearia sylvestris* AND scar | 7 |
| *Casearia sylvestris* AND wound healing | 12 |
| *Casearia sylvestris* AND anodyn | 0 |
| *Casearia sylvestris* AND cicatrix | 0 |
| *Casearia sylvestris* AND antinociceptive | 0 |
| *Casearia sylvestris* AND cicatrization | 0 |
| *Casearia sylvestris* AND wound closure | 1 |
| *Casearia sylvestris* AND anti-inflammator | 0 |
| *Casearia sylvestris* AND anti inflammator | 26 |
| *Casearia sylvestris* AND antiinflammator | 0 |

**Table S2.** Full patent list

| **Patent number** | **Title** | **Included? (Yes/No)** | **If not, why?** |
| --- | --- | --- | --- |
| WO202315364 | Cosmetic composition comprising *Casearia sylvestris*, *Schinus terebinthifolius*, *Hymenaea courbaril*, and *Lactobacillus*, and a method for preventing and/or treating signs resulting from skin aging. | No | Did not evaluate wound healing |
| Ep3545946 | Anti-senescence cosmetic composition comprising derivatives of *Casearia sylvestris* and/or *Hymenaea courbaril*, a method for preventing cellular senescence, and a cellular method for modulating the expression of β-galactosidase, p16, p21, and/or IL-8. | No | Did not evaluate wound healing |
| BRPI0602094 | Medicinal composition based on *Casearia sylvestris* and its therapeutic use. | Yes | - |
| BRPI0306167 | Process for obtaining extracts and active fractions of *Casearia sylvestris* and their applications. | Yes | - |
| EP3466407 | Composition for modulating genes responsible for general skin functions, method for modulating the expression of such genes, and use of a plant extract. | No | Did not evaluate wound healing |
| BR102016011816 | Composition for modulating genes responsible for general skin functions, method for modulating gene expression, and use of *Casearia sylvestris*, *Schinus terebinthifolius*, and *Paeonia albiflora*. | No | Did not evaluate wound healing |
| EP3038715 | Composition comprising guaçatonga extract and aroeira extract for preventing and/or treating skin aging. | No | Did not evaluate wound healing |
| BR102018008368 | Mouthwash solution based on *Melaleuca alternifolia* and *Casearia sylvestris* for the prevention and treatment of mucositis in patients undergoing chemotherapy. | Yes | - |
| BRPI0900645 | Extracts, active fractions, and/or isolated compounds of *Casearia sylvestris*, pharmaceutical formulations containing them, and their uses. | Yes | - |
| BR102018071621 | Effect of glycolic extract of *Casearia sylvestris* Swartz (guaçatonga) on wound healing in rats. | Yes | - |
| BR102020000001 | Process for obtaining an active fraction from the essential oil of *Casearia sylvestris* leaves (Sw.), active fraction 1–6, and reversal activity in a fluconazole-resistant strain. | No | Did not evaluate wound healing |
| WO202370178 | Composition and process for producing bioactive bioceramic membranes and synthetic hydroxyapatite bone grafts. | No | Did not evaluate wound healing |
| BR102016022682 | Composition for modulating genes responsible for general skin functions, method for modulating gene expression, and use of *Casearia sylvestris*, *Schinus terebinthifolius*, and hyaluronic acid. | No | Did not evaluate wound healing |
| BR102016015370 | Aqueous nanoemulsion from the essential oil of *Casearia sylvestris* leaves (Sw.) for viral inhibition against herpes simplex virus type I. | No | Did not evaluate wound healing |
| BR102016022152 | Differentiated process for supercritical extraction of *Casearia sylvestris*. | No | Did not evaluate wound healing |
| BR102014008978 | Medicinal composition with antibiotic, anti-inflammatory, and wound-healing activity. | Yes | - |
| EP3150213 | Medicinal composition with antibiotic, anti-inflammatory, and wound-healing properties. | No | Did not use *Casearia sylvestris* |
| BRPI0805322 | Compounds with cytomodulatory activity, formulations containing them, and process for their preparation. | Yes | - |
| Ep3466406 | Anti-wrinkle cosmetic composition, composition system, and method for cosmetic skin treatment. | No | Did not evaluate wound healing |
| EP3501491 | Cosmetic anti-sagging composition, use of the composition, and anti-sagging treatment method. | No | Did not evaluate wound healing |
| EP3501494 | Cosmetic anti-blemish composition, use of the composition, and method for anti-blemish treatment. | No | Did not evaluate wound healing |
| EP-916663 | Esculentin A (18β,19β-diacetyloxy-18α,19α-epoxy-3,13(16),14-clerodatrien-2-one) and Esculentin B (18β,19β-diacetyloxy-18α,19α-epoxy-3,12,14-clerodatrien-2β-isovaleryloxy-6β,7α-diol), process for their preparation, and their use in the manufacture of medicines. | No | Did not use *Casearia sylvestris* |
| BR102016011793 | Anti-aging cosmetic composition and composition system. | No | Did not evaluate wound healing |
| US9980998 | Medicinal composition with antibiotic, anti-inflammatory, and wound-healing activity. | No | Did not focus on *Casearia sylvestris* |
| WO202082140 | Processes for obtaining liquid concentrates at room temperature from plant species intended for infusion or decoction, as well as from wine, and products obtained and provided in individual portable doses. | No | Did not use *Casearia sylvestris* |
| WO2021191811 | Novel wound gel composition. | Yes | - |
| BR102016011821 | Composition for modulating genes responsible for general skin functions, method for modulating gene expression, and use of passion fruit ceramides and *Cichorium intybus*. | No | Did not use *Casearia sylvestris* |
| BR102015021047 | Improved liquid chromatograph for extraction of micromolecules from solid matrices and chromatographic analysis method using said equipment. | No | Not formulation relatated |
| WO202077428 | Compounds, their use in the preparation of a pharmaceutical composition, and a pharmaceutical composition comprising 7,11b-dihydro-6H-indeno[2,1-c]chromene-3,6a,9,10-tetrol, its derivatives or analogs, neutral or ionized, for prevention and/or senolytic therapy. | No | Did not use *Casearia sylvestris* |
| Ep2499123 | Anti-inflammatory compounds. | No | Did not use *Casearia sylvestris* |
| BR102017004926 | Biopolymers composed of polysaccharides and derivatives of cashew nut shell liquid. | No | Did not use *Casearia sylvestris* |
| BR102018077212 | Topical gel formulation with wound-healing activity containing microalgae extract. | No | Did not use *Casearia sylvestris* |
| BR102014028886 | Device for attracting or repelling insects and method for gradual release of a volatile substance using said device. | No | Did not use *Casearia sylvestris* |
| Ep2320879 | Pharmaceutical composition comprising jasmonates. | No | Did not use *Casearia sylvestris* |
| Ep2654665 | Method for developing a liquid composition to be applied to the skin as a foam and a composition suitable for topical application. | No | Did not evaluate wound healing |
| WO200667419 | Myrtaceous honey and its use as an immunomodulator. | No | Did not use *Casearia sylvestris* |
| US20100292280 | Antipyretic vasodilators. | No | Did not evaluate wound healing |
| WO201372332 | Methods for using bitter taste receptor antagonists. | No | Did not evaluate wound healing |
| Ep2986117 | Binary insecticidal or pesticidal mixture. | No | Did not evaluate wound healing |
| US20020194646 | Methods for inducing dwarf phenotypes in plants. | No | Did not evaluate wound healing |
| KR101401154 | Pharmaceutical composition for inhibiting regulatory T-cell activity comprising methyl gallate. | No | Did not evaluate wound healing |

**Table S3.** Raw data table used for patent analysis

| **Patent** | **Plant use** | **Final product** | **Pharmacological application** | **Analysis** |
| --- | --- | --- | --- | --- |
| BRPI0602094 | - | Ointment (orobase) | Healing/antiviral | Clinical analysis |
| BRPI0306167 | Purified extract, from leaves | Enhanced and more purified extract | Gastroduodenal ulcer | Chemical characterization of the active fractions |
| BR102018008368 | Extracts | Mouthwash solution | Mucositis treatment | Clinical analysis, patient wound test |
| BRPI0900645A2 | Extracts, active fractions and/or isolated compounds from *Casearia sylvestris* | Oral and intravenous dosage forms | Gastroduodenal ulcer | Chemical characterization of the active fractions |
| BR102018071621A2 | Extrato glicólico | Nano-spray/Biofilm | Wound healing | In vivo, wound area measurement and histopathological analysis |
| BR102014008978A2 | Extrato seco | Biofilm | Wound healing/antibiotic | Inhibition of microbial growth |
| BRPI0805322A2 | Purified extract, from leaves | Isolated extract of Casearin X | Cytomodulatoryn treatment of related wounds | In vivo, hystopathological analysis |
| WO2021191811 | - | Gel | Wound healing | Clinical analysis, patient wound test |

**Table S4.** Raw data table used for scientific analysis

| **Reference** | **Part of the plant** | **Extraction method** | **Final product** | **Pharmacological application** | **In vitro or In vivo** | **Type of cell** | **Species** | **Analysis** |
| --- | --- | --- | --- | --- | --- | --- | --- | --- |
| De Campos, 2015 | Leaf | Hydroalcoholic extraction | Biofilm and liquid formulation | Healing of burn injuries | In vivo | - | Adult male swiss and wistar rats | Macroscopic wound healing score (0–5) based on infection, inflammation, necrosis, and epithelialization; treatments: saline spray, extract spray, biofilm, and extract-loaded biofilm |
| De Mattos, 2007 | Dried leaf | Maceration | Hydroalcoholic extract | Antinociceptive | In vivo | - | Male swiss mice | Antinociceptive activity evaluated in mice treated orally with hydroalcoholic extract |
| Dezena, 2021 | Not reported | Percolation | Tincture | Wound healing | In vitro | Wistar rat skin | - | Microscopy (histology sample) |
| Lipinski, 2012 | Leaf and twigs | Decoction | Gel | Wound healing | In vivo | - | Beef cattle | Wound area measurement and histological analysis |
| Piovezan, 2017 | Leaf | Hydroalcoholic extraction | Ointment | Treatment of chronic post-ischemia pain | In vivo | - | Mice | Behavioral, histological and molecular |
| Napolitano, 2005 | Leaf, stem wood, stem bark, root wood and root bark | Maceration | Crude plant extract | Anti-inflammatory | In vitro | J774 murine macrophage cell line | - | Griess reagent assay |
| Trecco, 2014 | Leaf | Maceration | Natural latex membranes incorporated with ethanolic extract | Anti-inflammatory and wound healing | In vitro | Not reported | - | In vitro release analysis using UV spectroscopy |
